# Supplementary figures and images for: Incompatibility between two major innovations shaped the diversification of fish feeding mechanisms
Source: PLoS Biol. 2025 Jun 24;23(6):e3003225. doi: 10.1371/journal.pbio.3003225 (PMC12186908; doi:10.1371/journal.pbio.3003225)

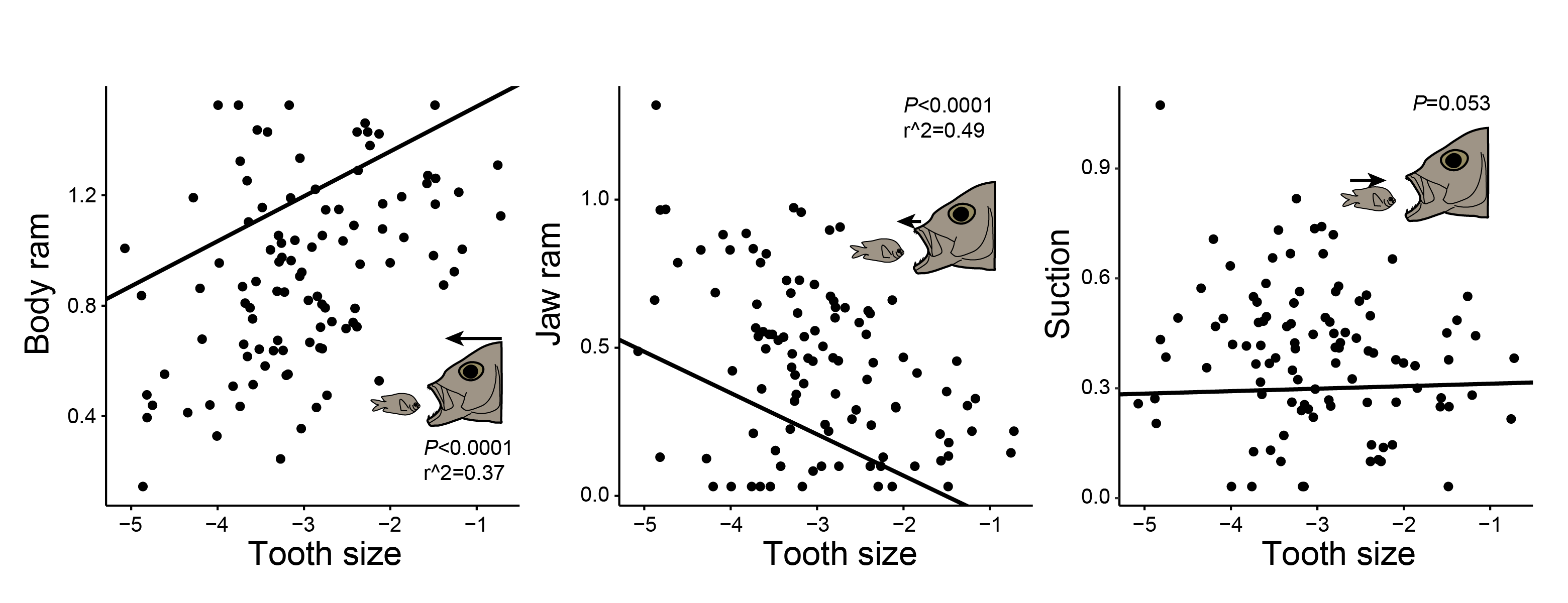

Supplement: S1 Fig — Phylogenetic generalized least-squares (PGLS) regressions between tooth size, quantified as a log-shape ratio, and the arcsine-transformed relative contributions of body ram, jaw ram, and suction, for 102 species of ray-finned fishes, after removing all 59 species of cichlids. P-values and r2pred are reported for each regression. The data underlying this figure can be found in S1 Data and S2 Data. (TIF) [file pbio.3003225.s001.tif]

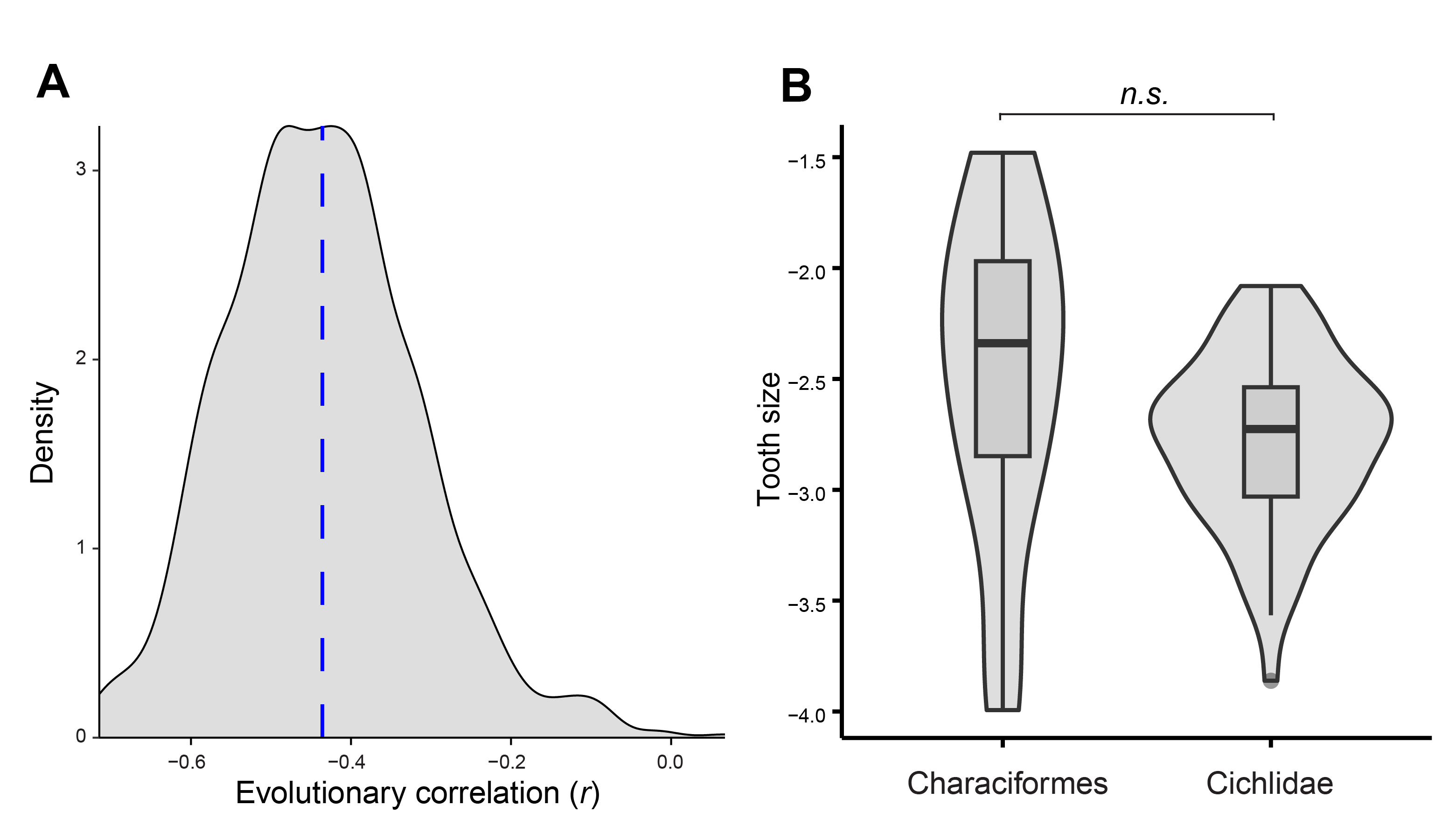

Supplement: S2 Fig — (A) Posterior distribution of the evolutionary correlation coefficient (r) between tooth size and body and jaw ram feeding, estimated under a threshold model. The blue dotted line indicates the mean of the distribution. (B) Clade comparisons of tooth size between Characiformes (no premaxillary protrusion) and Cichlidae (premaxillary protrusion). Significance is reported from Welch’s Analysis of Variance (ANOVA) test. The data underlying this figure can be found in S1 Data and S4 Data. (TIF) [file pbio.3003225.s002.tif]
