# Supplementary material for: Incompatibility between two major innovations shaped the diversification of fish feeding mechanisms
Source: PLoS Biol. 2025 Jun 24;23(6):e3003225. doi: 10.1371/journal.pbio.3003225 (PMC12186908; doi:10.1371/journal.pbio.3003225)
Supplement: S1 Table — A complete list of taxa included in this study. Species with exceptionally large teeth, defined as the upper 10% of the tooth size distribution, are marked with a (*). (PDF) [file pbio.3003225.s003.pdf]

S3 Table. List of taxa included in this study. A complete list of taxa included in this study. Species with exceptionally large teeth, defined as the upper 10% of the tooth size distribution, are marked with a (*).

Order	Family	Species
**Acanthuriformes**	**Acanthuridae**	*Acanthurus lineatus*
		Zebrasoma flavescens
	**Chaetodontidae**	*Chaetodon lunula**
		Chelmon rostratus
	**Haemulidae**	*Emmelichthyops atlanticus*
		Haemulon vitattum
	**Lobotidae**	*Datnoides microlepis*
	**Malacanthidae**	*Hoplolatilus purpureus*
	**Scatophagidae**	*Scatophagus argus*
	**Siganidae**	*Siganus vulpinus*
	**Zanclidae**	*Zanclus cornutus**
**Anabantiformes**	**Osphronemidae**	*Osphronemus laticlavius*
**Anguilliformes**	**Muraenidae**	*Gymnothorax griseus*
**Atheriniformes**	**Melanotaeniidae**	*Melanotaenia goldiei*
**Batrachoidiformes**	**Batrachoididae**	*Opsanus beta*
**Beloniformes**	**Belonidae**	*Xenentodon cancila*
**Blenniiformes**	**Blenniidae**	*Ecsenius midas**
	**Chaenopsidae**	*Chaenopsis alepidota*
	**Plesiopidae**	*Plesiops coeruleolineatus*
		Paraplesiops poweri
	**Pomacentridae**	*Chromis cyanea*
		Chromis viridis
		Hypsypops rubidundus
		Abudefduf saxatillis
		Stegastes fuscus
		*Microspathodon chrysurus**
**Carangiformes**	**Carangidae**	*Trachinotus blochii*
	**Latidae**	*Lates niloticus*
	**Sphyraenidae**	*Sphyraena barracuda*
**Centrarchiformes**	**Centrarchidae**	*Lepomis macrochirus*
		Lepomis gibbosus
	**Cirrhitidae**	*Oxycirrhites typus*
		Cirrhitus pinnulatus
		Paracirrhites arcatus
	**Kyphosidae**	*Kyphosus sectatrix*
	**Terapontidae**	*Terapon jarbua*
**Cypriniformes**	**Cyprinidae**	*Neolissochilus stracheyi*
		Leptobarbus hoevenii

**Cyprinodontiformes**	**Aplocheilidae**	*Aplocheilus lineatus*
	**Poeciliidae**	*Poecilia latipinna*
**Cichliformes**	**Cichlidae**	*Altolamprologus compressiceps*
		Aristochromis christyi
		Astatotilapia burtoni
		Astatoreochromis alluaudi
		Astronotus ocellatus
		Asprotilapia leptura
		Bathybates minor
		Buccochromis nototaenia
		Caquetaia krausii
		Chalinochromis brichardi
		Chilotilapia rhoadesii
		Cincelichthys bocourti
		Copadichromis borleyi
		Crenicichla regani
		Cyprichromis leptosoma
		Dimidiochromis compressiceps
		Ectodus descampsii
		Enterochromis paropius
		Etroplus maculatus
		Geophagus abalios
		Gnathochromis pfefferi
		Greenwoodochromis bellcrossi
		Harpagochromis "orange rock hunter"
		Heros severus
		Hemichromis elongatus
		Herichthys minckleyi
		*Julidochromis dickfeldi**
		Labidochromis caeruleus
		Labeotropheus trewavasae
		Lepidiolamprologus elongatus
		Lepidiolamprologus nkambe
		Lepidiolamprologus mimicus
		Limnotilapia dardenii
		Limnochromis auritus
		Limnochromis abeelei
		Maylandia callainos
		Myaka myaka
		Neochromis rufocaudalis
		Nimbochromis venustus
		Orthochromis stormsi
		Parachromis managuensis
		Paratilapia polleni
		Paraneetroplus gibbiceps
		Parachromis dovii
		Pelvicachromis pulcher
		Petenia splendida
		Perissodus microelpis
		Pterophyllum scalare
		Pundamilia pundamlia
		Pyxichromis orthostoma
		Rhamphochromis longiceps
		Simochromis babaulti

		Teleogramma brichardi
		Trematocara variabile
		Trichromis salvini
		Tropheus moorii
		Tyrannochromis nigriventer
		Xenotilapia rotundiventralis
	**Polycentridae**	*Monocirrhus polyacanthus*
**Characiformes**	**Acestroryhnchidae**	*Acestrorynchus microelpis*
	**Alestidae**	*Hydrocynus vittatus**
	**Anostomidae**	*Leporinus fasciatus**
	**Characidae**	*Charax gibbosus*
		Exodon paradoxus
		Gymnocorymbus ternetzi
	**Chilodontidae**	*Chilodus punctatus*
	**Chalceidae**	*Chalceus erythrurus*
	**Ctenoluciidae**	*Ctenolucius hujeta*
		Boulengerella maculata
	**Cynodontidae**	*Hydrolycus armatus**
	**Distichodontidae**	*Distichodus sexfasciatus**
	**Hemiodontidae**	*Hemiodus gracilis*
	**Hepsetidae**	*Hepsetus odoe*
	**Serrasalmidae**	*Piaractus brachypomus**
		Metynnis argenteus
**Elopiformes**	**Megalopidae**	*Megalops cyprinoides*
**Gobiiformes**	**Apogonidae**	*Glossamia aprion*
	**Butidae**	*Butis butis*
		Oxyleotris marmorata
	**Eleotridae**	*Dormitator lebretonsis*
	**Gobiidae**	*Stigmatogobius pleurostigma*
	**Microdesmidae**	*Ptereleotris microlepis*
		Ptereleotris evides
		Nemateleotris magnifica
**Labriformes**	**Labridae**	*Epibulus insidiator*
		Coris formosa
		*Scarus iseri**
**Lophiiformes**	**Antennariidae**	*Antennarius commerson*
**Osteoglossiformes**	**Mormyridae**	*Mormyrus longirostris*
	**Osteoglossidae**	*Osteoglossum bicirrhosum*
**Perciformes**	**Anthiadidae**	*Odontanthias borbonius*
		Pseudanthias dispar
	**Caesionidae**	*Pterocaesio pisang*
	**Epinephelidae**	*Paranthias furcifer*
		Cephalopholis urodeta
		Epinephelus ongus
		Cromileptes altivelis
		Cephalopholis miniata
		Plectropomus laevis
	**Gasterosteidae**	*Gasterosteus aculeatus*
	**Grammistidae**	*Rypticus maculatus*

	**Liopropomatidae**	*Liopropoma rubre*
	**Serranidae**	*Serranus annularis*
		Hypoplectrus puella
		Serranus tigrinus
	**Scorpaenidae**	*Inimicus didactylus*
		Synanceia verrucosa
		Pterois volitans
**Siluriformes**	**Pimelodidae**	*Sorubim lima*
		Pimelodus pictus
**Synbranchiformes**	**Mastecembelidae**	*Mastecembelus armatus*
**Syngnathiformes**	**Aulostomidae**	*Aulostomus maculatus*
	**Callionymidae**	*Dactylopus dactylopus*
	**Mullidae**	*Parupeneus cyclostomus*
**Tetraodontiformes**	**Balistidae**	*Pseudobalistes fuscus***
		*Rhinecanthus rectangularis***
	**Tetraodontidae**	*Canthigaster bennetti***
		*Sphoeroides spengleri***
