# Supplementary material for: Incompatibility between two major innovations shaped the diversification of fish feeding mechanisms
Source: PLoS Biol. 2025 Jun 24;23(6):e3003225. doi: 10.1371/journal.pbio.3003225 (PMC12186908; doi:10.1371/journal.pbio.3003225)
Supplement: S2 Table — Dirichlet regression model fitting using the ‘DirichReg’ function in the R package ‘DirichletReg’. ‘dr’ is the compositional data of the proportions of body ram, jaw ram, and suction, and is tooth size, corrected for body size. AIC scores, corrected for small sample size, are reported. The best-fit model is bold. (PDF) [file pbio.3003225.s004.pdf]

S2 Table. Dirichlet regression model fitting results. Dirichlet regression model fitting using the '*DirichReg*' function in the R package '*DirichletReg*'. 'dr' is the compositional data of the proportions of body ram, jaw ram, and suction, and is tooth size, corrected for body size. AIC scores, corrected for small sample size, are reported. The best-fit model is bold.

Model	Formula	Parameterization	AICc
model 1 (null)	dr ~ 1 | 1 | 1	common	-397.1422
**model 2**	dr ~ s	common	-441.4276
model 3	dr ~ s | 1	alternative	-414.4703
model 4	dr ~ s + s^2	alternative	-410.6331
model 5	dr ~ s + s^2 | s + s^2 | s + s^2	common	-439.3474
model 6	dr ~ 1 | s	alternative	-401.8757
