# Supplementary material for: Incompatibility between two major innovations shaped the diversification of fish feeding mechanisms
Source: PLoS Biol. 2025 Jun 24;23(6):e3003225. doi: 10.1371/journal.pbio.3003225 (PMC12186908; doi:10.1371/journal.pbio.3003225)
Supplement: S3 Table — Results of continuous trait evolution model fitting with ‘OUwie’. AIC scores, corrected for small sample size, are reported as the mean of 100 fitted models. The best-fit model (OUM) is identified in bold. Parameters refer to the Brownian rate parameter (σ2), strength of selection (α) and location of the adaptive peak (θ). (PDF) [file pbio.3003225.s005.pdf]

S1 Table. Ornstein-Uhlenbeck model fitting results. Results of continuous trait evolution model fitting with '*OUwie*'. AIC scores, corrected for small sample size, are reported as the mean of 100 fitted models. The best-fit model (OUM) is identified in bold. Parameters refer to the Brownian rate parameter (σ^2), strength of selection (α) and location of the adaptive peak (θ).

Model	Parameters	AICc
BM1	single σ^2	357.52
BMS	multiple σ^2	359.17
OU1	single σ^2, single α, single θ	342.25
**OUM**	**single σ^2, single α, multiple θ**	**335.32**
OUMV	multiple σ^2, single α, multiple θ	336.84
OUMA	single σ^2, multiple α, multiple θ	336.06
OUMVA	multiple σ^2, multiple α, multiple θ	337.27
