# Supplementary material for: Incompatibility between two major innovations shaped the diversification of fish feeding mechanisms
Source: PLoS Biol. 2025 Jun 24;23(6):e3003225. doi: 10.1371/journal.pbio.3003225 (PMC12186908; doi:10.1371/journal.pbio.3003225)
Supplement: Attachment — Submitted filename: response_to_reviewers.docx [file pbio.3003225.s013.docx]

**Note: Line numbers correspond to the document with tracked changes.**

**Reviewer #1:**

Review for Peoples, N. paper

**Referee Comment:** Overall, I liked the paper and the analysis. I think this is a very interesting idea and topic and I'm glad the authors are tackling it. It is very well written, and the statistics are sound. However, I'm a little concerned about the methodology for measurements of tooth size. Comparing the largest tooth in the jaw no matter its location is concerning. I wonder if the authors were to test their theory on the size on the incisor across all species or just the size of the molars if the same relationships would be found. I think this is a worthwhile paper, I'm just concerned there may be a sampling bias based on the methods of measuring the tooth size. Would the authors be able to run an analysis on just incisor or molar size across all species? I'm also wondering about average tooth size since there's a lot of heterodonty in fishes. Would the authors find the same relationship if they looked at average tooth size? You could also look at the range in tooth size as being an indicator. These are some concerns and questions I have about the paper.

**Authors’ Response:** Thank you for your positive evaluation of our manuscript. We have addressed each of the comments below and as a result, believe the manuscript has improved greatly. We understand your concerns about our usage of largest tooth size, however, we believe that measuring the single largest tooth is appropriate for this analysis for a few reasons. First, while mammals often have easily distinguishable tooth classes (e.g. incisors, molars, canines), this is not the case in ray-finned fishes, which are often morphologically homodont. While comparing results between incisors and molars may be interesting for a study of mammalian dentitions, we believe it would not be appropriate for our study because it is not possible to make a consistent distinction between these discrete tooth types in fishes – neither by tooth type nor location. We also note that for most fishes examined in this study (and in our observations generally), the largest tooth is often found at the anterior end of the jaws, so we would anticipate that the location along the jaw is actually relatively consistent a across our phylogenetic sampling. Additionally, while tooth position along the jaw may affect lever ratios with implications for the transmission of force to a bite (see Coehn et al. 2020, J of Anatomy and Mihalitsis & Bellwood 2019, R. Soc. Open Science), there is no evidence to suggest that location of large teeth along the jaw might influence the relative contributions of body ram/jaw ram/suction. Further, the largest tooth in the jaws of a predator will primarily be the tooth making contact with the prey upon capture, thus making it the most functionally relevant.

We agree that fishes often have variation in tooth size within the jaw, and indeed many fishes with large teeth also have numerous small teeth. However, small teeth are unlikely to introduce any constraint or reflect any tradeoff, as our results show that species with small teeth can use any combination of body ram, jaw ram, and suction (Fig. 2). In contrast, the presence of just a single or few large teeth is likely sufficient to introduce biomechanical constraints and often reflects transitions to new feeding strategies (e.g. spearing behavior in *Hydrolycus armatus*), which aligns with the view that large teeth are an innovation across fishes when they arise. For example, just a single large tooth would place a requirement on available space in the dentigerous bone for replacement, regardless of whether additional smaller teeth were present. We thus believe measuring the single largest tooth is appropriate in the context of our study, and that analyzing average tooth size would lead to less biologically-meaningful and interpretable relationships.

**Referee Comment:** Lines 65-66: "Fish primarily capture prey by using a combination of forward swimming, termed 'ram', and suction…" I would use this sentence to describe the difference between body ram and jaw ram. Having this definition in the intro would be helpful

**Authors’ Response:** This is a great suggestion – we have now included these definitions of body ram, jaw ram, and suction earlier in the introduction (lines 73-75).

**Referee Comment:** Lines 62-87: I feel as if this paragraph jumps around and is not as fluid as it could be. I think starting off with describing the prey capture then moving to teeth then moving back to suction is confusing. I would just talk about prey capture strategies and then talk about teeth. Or maybe start with teeth and then talk about suction and ram

**Authors’ Response:** Thank you for this suggestion, which is something the second reviewer commented on as well. We have reorganized this section considerably to first discuss teeth and then suction and jaw protrusion. We believe that this organization improves the clarity considerably. For the updated version of this paragraph, please see lines 62-90.

**Referee Comment**: Line 179: Not sure if "shape ratio" is ever defined in the paper.

**Authors’ Response:** Apologies if this was not clear. The “shape ratio” reported here refers to the size-corrected tooth length (e.g., the tooth length divided by the geometric mean of standard length, upper jaw length, and jaw width). This is defined in the methods (lines 413-414). We use the geometric mean of three measurements to capture fish ‘size’ because body shape varies considerably across the sample of species included in our study (See Price et al. 2019 Integrative & Comparative Biology)

**Referee Comment:** Line 223: Just a thought but maybe you could talk about the drag teeth may incur on the slender jaws of fish that have protrusible jaws? Not a necessity but thought it might be a cool avenue to think about.

**Authors’ Response:** This is an interesting thought, and we agree that larger teeth could increase drag during a jaw ram strike. We have included mention of this in lines 236-238.

**Referee Comment:** Figure 2a: what does the shaded area and dotted lines represent?

**Authors’ Response:** The shaded areas and dotted lines were meant to highlight the bounds of the distribution. Since they do not specify any statistical limit (e.g. confidence intervals) we removed them to avoid confusion. The updated figures do not have these shaded areas.

**Reviewer #2:**

**Referee Comment:** I applaud the authors on a really interesting and compelling read. I can't recall that many papers that look at this sort of "interference" between trait sets. Cool stuff. My comments and critiques are mostly minor and mostly cover times where I think the authors could do a better (meaning more clear) job of presenting what they are doing and why. I leave it to the Editor(s)' discretion about whether the concerns below merit revision, etc.

**Authors’ Response:** Thank you for your positive comments and evaluation of our manuscript. Please find each of your comments addressed below.

**Referee Comment:** Lines 62-87: I understand what the authors are doing, but this paragraph comes across a bit stilted ie with short factual statements repeated without a whole lot timing them all together. Perhaps another approach is a paragraph on teeth, then a paragraph on suction/ram feeding (or the reverse), then a synthesis.

**Authors’ Response**: Thank you for this suggestion, which is something the first reviewer commented on as well. We have reorganized this entire section to first discuss teeth followed by suction and jaw protrusion. We believe that this organization improves the clarity considerably. For the updated version of this paragraph, see lines 62-90.

**Referee Comment:** Lines 131-136: I like this idea of comparing two extremes, like cichlids and characiforms, but I wonder if it's a little too extreme of an example. Is it worth incorporating a third clade with jaw kinesis, but with variability in kinesis within the clade (wrasses?)? Comparing cichlids to characiforms is like comparing apples to cucumbers. Moreover, while other parts of the text correct for sample size differences, does this section?

**Authors’ Response:** We agree that it would be interesting to include additional clades that vary in their jaw kinesis. Unfortunately, we do not have a third large clade with variable kinesis such as wrasses in our dataset, which limits us to a comparison between cichlids and characiformes. However, we do believe this comparison highlights an interesting point – that teeth may generally be larger in clades that lack protrusion.

Thank you for pointing out the need to account for sample size differences. We used Levene’s tests to assess if the group variances were unequal (which they were, line 470), and Welch’s ANOVA to make this comparison while accounting for the unequal group variances (lines 138-140).

**Referee Comment:** Lines 133-134: I believe some characiforms and cithariniforms do have independently derived jaw protrusion, like the halftooths. See Gery 1962.

**Authors’ Response**: Thanks for mentioning this. We have clarified that “characiformes….of which most species do not have premaxillary jaw protrusion” (line 136) to reflect the independent origin of jaw protrusion in *Bivibranchia* (Gery 1962; Vari 1985).

**Referee Comment:** Lines 154-157: the authors should explain why they took this binning approach in the first place - what question is this method answering and what should readers expect to find. You could do this by starting the paragraph with a question that sets up the rest of the prose.

**Authors’ Response:** This is a great suggestion to improve the clarity of this analysis. We have edited the first two sentences of this paragraph to better state why we have taken this binning approach. This section now reads (lines 159-162); “The functional diversity of prey capture for lineages with large teeth may be limited if large teeth constrain species to jaw ram feeding. We therefore, quantified the diversity of prey capture for a range of tooth sizes, and measured the variance in body ram, jaw ram, and suction contributions for 10 equal-frequency tooth-size bins”

**Referee Comment:** Lines 166-167: this is presumably an issue of formatting these sorts of papers with Results prior to Methodology - but it's not clear to me at this point how the authors measured "diversity of kinematic strategies" unless by strategies they just mean suction, body ram, and jaw ram.

**Authors’ Response:** Thank you for pointing this out, as we do indeed mean suction, body ram, and jaw ram as the diversity of kinematic strategies. We have specified “…using a greater diversity of kinematic strategies by utilizing varied amounts of jaw ram and suction” to make this point clearer (lines 174-175).

**Referee Comment:** Lines 182-183: explain to readers what the authors mean when they say: "threshold model for evolution."

**Authors’ Response**: We agree that additional information about the threshold model would be beneficial here. We included more explanation about what the threshold model represents and how we interpret the negative evolutionary correlation that we recover (lines 190-196).

**Referee Comment:** Lines 212-213: I do not think that tooth replacement is most frequently intraosseus in teleosts, my recollection is that this is actually quite rare, thinking of papers by Bemis and colleagues. When looking at the references the authors cite for this, both papers examine single species. So, I couldn't confirm, based on citations by the authors, that this is a factual statement. Perhaps the authors, with a focus on cichlids and characiforms, assumed intraosseus replacement is found more often than it is?

**Authors’ Response:** Thank you for pointing this out. We have clarified this to mention that intraosseous is the primary replacement mode among species in our study (lines 224-225) and included a reference (Trapani 2001) to support this. Additionally, we included mention that intraosseous development is a derived state amongst teleosts (lines 223-224).

**Referee Comment:** Lines 224-226: I guess I missed it at some point - but how are the authors defining "large" teeth vs. "small" teeth?

**Authors’ Response:** We define large teeth as species within the upper 10% of the tooth size distribution (line 241). We have now moved this explanation further up in our manuscript to improve clarity. These species are marked with an asterisk in Table S1.

**Referee Comment:** Lines 224-261: I found this to be an interesting section, but it spends quite a bit of time discussing data that the authors themselves didn't collect. Recommend condensing this to some pertinent examples for a more concise section. Moreover, several times claims are stated about the morphology or ecology of one fish group or another, but aren't referenced. The authors cite Berkovitz & Shellis' book on teeth quite frequently, as a bit of a catch-all reference, without

**Authors’ Response:** We agree that this section could benefit from being condensed to pertinent examples. We have done so and included a limited number of relevant examples (lines 252-273). Additionally, we condensed the writing within this section and included additional references (lines 246-247) to support these examples regarding a species’ ecology/morphology (e.g. herbivory, frugivory).

**Referee Comment:** Lines 268-269: I understand what the authors are saying about "negative implications for resource processing" but again, the authors didn't actually quantify deficits in prey processing, so it seems like a bit of a reach. I imagine that many of these fishes might actually not suffer any deficits, if they have capable pharyngeal jaws. I acknowledge that's dodging a constraint through use of an additional tool, but worth mentioning the caveat.

**Authors’ Response**: We have changed this mention to “resource acquisition abilities” to better reflect that idea that jaw ram feeders with small teeth may not be able to remove attached resources (e.g. turf algae) or damage/restrain prey before intraoral transport (line 294).

**Referee Comment:** Figure 1: examining this figure, I wonder if the sampling strategy here included enough species on the jaw ram or suction end of this ternary plot to make a meaningful comparison. Or if those species exist in the first place. If the latter is the case, then that would invoke some other discussions of constraint perhaps?

**Authors’ Response:** Previous research (Longo et al. 2016, J. Experimental Biology) concluded that body ram, not suction and jaw ram, is the primary axis of prey capture diversity in fishes, and our results appear to confirm this even after expanded sampling. We made efforts to sample species that may have high jaw protrusion or suction but find that body ram remains the main axis. We acknowledge that our sample of species, while exceptionally large for a dataset that involves laboratory high-speed video, may not perfectly reflect the balance of feeding modes across ray-finned fishes generally. Nevertheless, we feel that we have sampled a number of extremes and a phylogenetically diverse group of speces.

**Referee Comment:** Figure 4: this is certainly a preference thing, but these cleared and stained jaws with teeth should be edited to that they have similar saturation, hue, contrast, color-balance, etc. The pufferfish image has a large grey blotch on one side. This figure could be refined with little effort and a bit of time in Photoshop.

**Authors’ Response**: Thank you for this feedback. We have edited this figure to improve its presentation.

**Referee Comment:** Lines 347-351: "We quantified the proportions of these three movements (body ram, jaw ram, suction) to closing the total distance between predator and prey" - how, specifically? Meaning, how did the authors differentiate among these three components for measurements? (Note: I found this later, lines 367-370… perhaps this section should be moved up). However, I'm still curious how the authors managed to distinguish these three components independent of each other.

**Authors’ Response**: The three components measured (body ram, jaw ram, suction) were all measured independently, meaning none of the components depended upon the position of the other. We chose sequences in which the camera did not move between frames; this allowed for direct measurement of distances between frames. The measure of suction is independent of body and jaw ram, as suction was measured using only the distance traveled by the prey. Body ram was measured as the distance between the center of the eye between frames. Importantly, jaw ram was measured from the “capture” frame; this means that the measure of jaw ram is independent of the amount of body movement between the “initiation” frame and “capture” frame.

**Referee Comment:** Lines 352-354: how did authors control or account for differences in kinematic sequences if prey were not kept consistent across studies?

**Authors’ Response**: In order to elicit feeding strikes from a wide range of taxa spanning many different ecologies, we used three different prey types, as not all species would feed on live feeder fish. We recognize that this could introduce some variation in our data, specifically regarding the effort of the strike (lines 380-381).

**Referee Comment:** Lines 356-359: was any randomization or inter-rater reliability done to see what the effect of measuring the "best" strike was? It seems to me that with this much leeway over what data the authors were analyzing, there can be some inherent bias in selection of which videos were deemed appropriate for analyses.

**Authors’ Response:** All videos of strikes were chosen for analysis by a single author (N.P.). We have added a sentence to clarify this in the methods (lines 387-388)

**Referee Comment:** Lines 377-391: is the largest tooth really the best option here? Rather than the most anterior or average sized tooth? How might the selection of a different metric of tooth size alter the results?

**Authors’ Response**: Please see our above response to Reviewer 1’s comment for a detailed explanation of our rationale for this decision.

**Referee Comment:** Lines 406-415: the authors mention high phylogenetic signal in the data, but the Dirichlet analyses are reported as not being able to account for this structure in the data. The authors should discuss the limitations of this approach.

**Authors’ Response:** Thank you for this comment. We note that the high phylogenetic signal refers specifically to tooth size alone. The purpose of the Dirichlet regression was to account for non-linear behavior that may affect the relationship between tooth size and feeding kinematics. We have specified this in the text (line 151). The PGLS analyses indicate significant relationships between tooth size and body/jaw ram while accounting for phylogenetic relatedness. This suggests that the relationship between tooth size and body/jaw ram is decoupled from phylogenetic relationships. A strong relationship, but not-significant PGLS result, would indicate that this relationship may be largely driven by phylogeny. Because we do not see evidence for this, we are more confident that the patterns uncovered using Dirichlet regression, which mirror those of PGLS, would not change if it was possible to account for phylogenetic relatedness in Dirichlet regression (lines 439-443). Additionally, we emphasize the results of PGLS, rather than the Dirichlet regression, in the main text.

**Referee Comment:** I always find it an interesting exercise to gauge how much of the literature in a manuscript is composed of self-citations. My rough estimate puts 30% of the papers cited in this manuscript as being the product of at least one of the authors.

**Authors’ Response:** Thank you for pointing this out, and we have made efforts to include a more diverse range of supporting references.
